# Implementing drug abuse treatment services in criminal justice settings: introduction to the CJ-DATS study protocol series

**DOI:** 10.1186/2194-7899-1-5

**Published:** 2013-11-18

**Authors:** Lori J Ducharme, Redonna K Chandler, Tisha RA Wiley

**Affiliations:** National Institute on Drug Abuse, 6001 Executive Blvd., Rm 5185 MSC 9589, Bethesda, MD 20892-9589 USA

## Abstract

**Background:**

Despite a growing pipeline of effective clinical treatments, there remains a persistent research-to-practice gap in drug abuse services. Delivery of effective treatment services is especially lacking in the U.S. criminal justice system, where half of all incarcerated persons meet the need for drug abuse or dependence, yet few receive needed care. Structural, financial, philosophical and other barriers slow the pace of adoption of available evidence-based practices. These challenges led to the development of a multi-site cooperative research endeavor known as the Criminal Justice Drug Abuse Treatment Studies (CJ-DATS), funded by the National Institute on Drug Abuse (NIDA). CJ-DATS engages university-based research teams, criminal justice agencies, and community-based treatment providers in implementation research studies to test strategies for enhancing treatment service delivery to offender populations.

**Methods/Design:**

This Introduction reviews the mission of NIDA, the structure and goals of the CJ-DATS cooperative, and the implementation studies being conducted by the participating organizations. The component Study Protocols in this article collection are then described.

**Discussion:**

CJ-DATS applies implementation science perspectives and methods to address a vexing problem – the need to link offender populations with effective treatment for drug abuse, HIV, and other related conditions for which they are at high risk. Applying these principles to the U.S. criminal justice system is an innovative extension of lessons that have been learned in mainstream healthcare settings. This collection is offered as both an introduction to NIDA’s work in this area, as well as a window onto the challenges of conducting health services research in settings in which improving public health is not the organization’s core mission.

## Background

The National Institute on Drug Abuse (NIDA), a component of the National Institutes of Health, US Department of Health and Human Services, supports a wide range of research to improve the detection, prevention, and treatment of drug abuse and addiction. The Institute’s research agenda includes developing evidence-based preventive interventions, behavioral treatments, and pharmacotherapies targeting drugs of abuse for the full range of patient populations. NIDA’s research agenda also includes identifying effective strategies to ensure that behavioral treatments and pharmacotherapies are successfully adopted, delivered, and sustained within a variety of settings providing services to individuals struggling with addictive disorders. Despite a growing pipeline of effective clinical treatments, there remains a persistent research-to-practice gap in drug abuse services. Implementation science is a relatively new area of focus for NIDA, and provides much-needed opportunities to develop effective strategies for bridging this service gap. NIDA’s Criminal Justice Drug Abuse Treatment Studies (CJ-DATS) cooperative fields multiple implementation research studies to promote uptake of treatment services for criminal offender populations. This article introduces a collection of study protocols emanating from this signature initiative, describes the urgency of the need for effective implementation strategies in this area, and identifies challenges and opportunities for conducting implementation science in criminal justice settings.

### Opportunities for drug abuse implementation science in criminal justice settings

An estimated 7 million adults in the United States are under some form of criminal justice supervision (Glaze & Parks 2012) and at 743 persons per 100,000 population, the US incarceration rate was the highest in the world as of 2009 (International Centre for Prison Studies (ICPS) 2009). Criminal justice involvement rates are driven in part by a large number of drug-related offenses. Indeed, half of all individuals incarcerated in US prisons and jails meet the criteria for drug abuse or dependence and could benefit from treatment (Chandler et al. 2009), but less than 20% actually receive an intervention (Karberg & James 2005; Mumola & Karberg 2006). Other health conditions related to drug use and addiction are also disproportionately high among those involved in criminal justice. Rates of smoking among inmates are twice those of the general population (Binswanger et al. 2009). It is estimated that 21% of all US residents who are living with HIV, 33% of those living with the hepatitis C virus, and 40% of those living with tuberculosis pass through a correctional facility on an annual basis (Hammett et al. 2002; Spaulding et al. 2009). Together, these data suggest that the US criminal justice system provides a prime, but often missed, opportunity for delivery of treatment services for drug abuse, HIV, and other related conditions.

The US criminal justice system is complex and multi-layered, and gaps in drug abuse service delivery appear in different forms and demand an array of solutions. NIDA’s implementation research portfolio in this area is particularly focused on the service needs of offenders at or nearing the point of re-entry to the community. In 2011, there were nearly 5 million adults on probation or parole in the US (Glaze & Parks 2012). Drug-related criminal charges, drug abuse, HIV and other related conditions are significant problems in community correctional populations, but these settings rarely have the capacity to deliver treatment services even when clients are properly screened and assessed. Moreover, probation and parole agencies are often ill-equipped to refer clients to appropriate and available community-based drug treatment and other healthcare services. Offenders re-entering the community are especially vulnerable to gaps in service delivery that arise from a lack of coordination between the criminal justice system and the health care, substance abuse treatment, public health, and social services agencies in the community.

A robust portfolio of research findings accumulated over the past two decades has established the effectiveness of drug treatment in reducing drug use and associated criminal activity (Inciardi et al. 1997; Pearson & Lipton 1999). NIDA synthesized much of this research into a document, *Principles of Drug Abuse Treatment for Criminal Justice Populations,* which provides a summary of the components of a high quality service delivery system for drug abuse treatment (Fletcher & Chandler 2006). But evidence-based practices and programs have been slow to be adopted within criminal justice agencies (Taxman & Belenko 2012). Dissemination and implementation are stymied by a number of structural challenges (Farabee et al. 1999; Linhorst et al. 2001). Criminal justice agencies are often isolated from clinical practice and from scientific research, slowing dissemination of evidence. Moreover, structural barriers to change can be daunting. Agency funding is cyclical, often unpredictable, and subject to sudden cuts in response to broader state and Federal budget crises and varying public support; as a result, the resources to support system-wide practice change can range from austere to uncertain at best. Within these organizations, management practices generally provide few incentives for innovation; there is limited accountability for program outcomes; and high staff turnover challenges the sustainability of new practices (Welsh & Harris 2008).

Together, the high rates of drug abuse, HIV, and related conditions among offender populations; the availability of evidence-based practices to address these needs; and the structural barriers to innovation make the US criminal justice system a ripe target for implementation science research. Needed are evidence-based strategies for changing the business practices of organizations and systems to fully integrate drug abuse treatment services for drug-involved individuals under criminal justice supervision, in a manner that respects and supports dual goals of public health and public safety.

## Methods/Design

In 2002, NIDA established a multisite cooperative research program known as the Criminal Justice Drug Abuse Treatment Studies (CJ-DATS). A total of 9 academic research centers, each with multiple criminal justice agency partners, were funded to develop and test integrated approaches to the treatment of offenders with drug use disorders. Projects were undertaken in 8 study areas including: screening and referral for drug abuse, mental health, and criminal risk problems; modifying treatment programs and interventions for reentering offenders; improving treatment engagement and retention; linking services in the community; and services to address the needs of special populations (Wexler & Fletcher 2007). While these studies yielded several effective service delivery models, each also identified a number of barriers to participating agencies’ continued use of these processes beyond the active study period.

A second phase of CJ-DATS, engaging a new cohort of research centers and agency partners, was launched in 2008 with a focus on conducting implementation research in these settings. Specifically, NIDA charged the cooperative with testing implementation strategies that could result in sustained uptake and delivery of services in three domains: delivery of medication-assisted treatment for offenders transitioning to the community; delivery of an HIV continuum of care (i.e., screening and counseling, risk reduction interventions, and continuity of antiretroviral treatment from prison or jail into the community); and implementation of screening and assessment processes to identify offenders with drug abuse and related health problems and to inform their treatment planning and re-entry process. In each domain, grantees were to focus on organizational and system-level implementation strategies, and to engage both community corrections and community-based treatment providers in a process that would leverage key facilitators, address barriers, and jointly address the public safety concerns of criminal justice agencies with the public health goals of the Institute and the community-based treatment partners.

NIDA’s ultimate goal for CJ-DATS is to identify implementation strategies that maximize the likelihood of sustained delivery of evidence-based practices to improve offender drug abuse and HIV outcomes, and to decrease their risk of reincarceration. These studies have proven challenging given the unique nature of the US criminal justice system, agencies’ multiple competing demands and limited resources, and the novelty of bringing implementation science to bear on these issues. Unlike traditional health care settings, the criminal justice organizations’ primary focus is not on offender health outcomes but on punishment, reform, and ensuring public safety. Likewise, individual offenders do not encounter these settings willingly, nor is receipt of health care services their primary concern. A review of all study protocols published in *Implementation Science* to date revealed no others examining implementation processes within criminal justice settings. This article collection is intended to provide insights into both the opportunities and challenges – for both implementation science and public health – that characterizes research in this venue.

### Articles in this collection

The implementation projects undertaken within the CJ-DATS cooperative reflect the research challenges and practical constraints of effecting change within large bureaucratic structures such as the U.S. criminal justice system (Gordon et al. 2011). All of these studies are guided by Proctor et al.’s (Proctor et al. 2009) conceptual framework in terms of measuring key outcome domains. The studies each examine one or more implementation outcomes (i.e., feasibility, fidelity, penetration, acceptability, sustainability, uptake, and/or costs) and service outcomes (generally service delivery and/or receipt). Where possible, sustainability beyond the active intervention phase is also being measured. Ultimately, the critical question is whether these implementation strategies result in lasting changes to agency practices, and the delivery of evidence-based drug treatment services to offenders re-entering the community.

Four study protocols are included in this collection. Three of the study protocols address issues of system coordination between community corrections agencies and community-based drug abuse treatment providers. These studies target three known, high-priority deficiencies in existing service coordination for offenders at re-entry: linkages to medication-assisted treatment, delivery of a continuum of care for HIV, and use of effective screening and assessment protocols for treatment planning. These protocols are designed to improve cross-agency collaboration, and to thereby improve drug abuse and/or HIV service delivery for persons in a system that is not resourced to deliver those services. Such problems are most effectively addressed not by asking criminal justice agencies and their staff to take on additional responsibilities and costs, but rather to initiate or improve their coordination with existing service providers. Likewise, if offenders must navigate multiple uncoordinated systems, their risk of relapse and recidivism is greater. Enhancing coordination among systems can create synergistic results that address both public health and public safety goals simultaneously. The fourth CJ-DATS study protocol engages corrections agencies in locally-specific and appropriate adaptations of an evidence-based clinical intervention (contingency management) to promote its use and sustainability.

Each of the studies employs a multifaceted implementation strategy (Powell et al. 2012; Grimshaw et al. 2001), including some combination of training, facilitated change teams, strategic planning, feedback, rapid cycle testing, coaching and/or technical assistance. In so doing, the four studies each address the Plan, Educate, Restructure, and Quality Management domains of Powell et al.’s taxonomy (Powell et al. 2012). (While the implementation strategies do not directly address Powell et al.’s other 2 domains (Financing, Policy), the change teams or strategic planning groups in several of the individual study sites may opt to attend to these domains in the process of engaging in the implementation intervention). Given NIDA’s recent investment in the specification and testing of the NIATx change process (Hoffman et al. 2012; McCarty et al. 2007), elements of this approach are common across the studies. To account for significant local variation among criminal justice settings, as well as to maximize the likelihood of long-term sustainability, each of the studies encourages sites to identify and prioritize their own service needs within the broad parameters of the protocol topic, and to focus on these in executing their implementation process. Likewise, each protocol engages key decision makers at each study site, and where possible builds-in obtaining their approval at critical junctures of the project before proceeding.

Each of the studies engages a team of corrections and treatment staff in a facilitated process to integrate one or more new services or processes. The degree of facilitation varies substantially across protocols, yielding a cross-study opportunity to examine the relationship between the degree and type of facilitation and the implementation outcomes. At one extreme, facilitation is entirely internal to the participating organizations; at the other extreme, an independent external coach is hired to work with each of the appointed change teams. In all sites, the research team provides project monitoring and data collection, and study-specific logistical support. These protocols are therefore testing whether facilitation or coaching of interorganizational change teams – strategies that have been used successfully in industry as well as in health care (Aarons et al. 2009; Loftus-Hills & Harvey 1999; Stetler et al. 2006) – can be adapted and deployed as effective implementation processes in criminal justice environments. This aspect is both a challenge and potential contribution of these protocols.

### Implementing Linkages to Medication-Assisted Treatment

The US Food and Drug Administration (FDA) has approved medications for use in the treatment of alcohol and opiate dependence. Unlike much of the healthcare system, in which new medications are routinely introduced and adopted, the US addiction treatment system has been slow to adopt pharmacotherapies (Knudsen et al. 2011), lacks trained and credentialed personnel to deliver them (Knudsen et al. 2010), and evidences a longstanding philosophical preference for “drug free” treatment (Knudsen et al. 2005; Pollack & D’Aunno 2008). These constraints and concerns are magnified in community corrections agencies, where the appropriate role of pharmacotherapy is not well understood, and the medically appropriate use of opioid agonists (e.g., methadone) may present challenges for monitoring offenders’ illicit drug use. In the protocol, “Medication-Assisted Treatment Implementation in Community Correctional Environments (MATICCE)”, community corrections staff engage in training about addiction pharmacotherapies, while leadership in the corrections and treatment facilities engage in a joint strategic planning process to identify and resolve barriers to efficient flow of clients across the two systems. Importantly, the objective is to leverage treatment services that *already exist* in the community. This means that the implementation strategy is not designed to promote the delivery of clinical services nor medication prescribing *within or by* probation and parole offices, but rather is targeted at facilitating linkages between organizations that, despite sharing the same clients, lack incentive to coordinate services, or otherwise do not view themselves as sharing a common mission.

### Implementing an HIV continuum of care

Evidence-based approaches exist for HIV risk reduction, testing, and treatment with antiretroviral therapy. However, there are significant gaps in this service continuum for offenders in the criminal justice system. Services may or may not be provided while individuals are incarcerated; transitions to the community may introduce additional service disruptions; and individual offenders face challenges navigating their prevention, testing and treatment options. This study protocol tests a modified NIATx process (McCarty et al. 2007) to facilitate site-specific improvements in the HIV services continuum. In both the control sites and those sites randomized to the modified NIATx process, criminal justice staff receive training on the fundamentals of HIV prevention and treatment. Sites randomized to the experimental condition form a local change team to engage in a process improvement approach with external coaching to implement a more complete HIV services continuum. Within the overall parameters of the protocol – which emphasizes HIV testing and linkage to treatment – sites assess local needs and existing services, set priorities for service improvements, and develop specific goals and strategies for achieving them. The protocol in all sites targets three goals: improving the perceived value of HIV services among corrections staff; increasing service penetration for inmates with or at risk for HIV; and improving the quality of HIV service delivery.

### Implementing assessment and treatment planning processes

This protocol also uses a modified NIATx approach, but rather than focus on promoting the uptake of specific services, it engages corrections and treatment agencies to improve the quality of interagency communication through the effective use of assessment and case planning processes and treatment referrals. Both interagency and intra-agency change processes are targeted. A multi-phase implementation protocol is used, wherein agencies engage in team development, needs assessment, planning, implementation, and sustainability in distinct steps. Each site’s change team has a designated facilitator, and their activities are coordinated across sites through the use of a facilitator manual and ongoing fidelity measurement. Early and delayed-start sites allow the research team to control for effects of environmental changes within states. The key outcomes for this protocol reflect improvements in the use of assessment and case planning procedures for offenders. Specifically, offenders should not only receive comprehensive assessments, but the results of these assessments should inform case plans, and the information in the case plan should be conveyed to the local treatment provider agency to which the offender is referred upon release. The protocol targets critical communications channels between otherwise highly segregated correctional and treatment agencies.

### Implementing contingency management in correctional settings

The fourth study protocol in this collection reflects a more traditional implementation problem – the need to integrate a specific evidence-based practice into an organization’s routine business processes. The implementation target for this protocol is contingency management, a structured incentive program for rewarding individual achievement of prescribed behavioral goals. Contingency management has a well-established evidence base in substance abuse treatment settings (Hartzler et al. 2012), but requires modification for use in criminal justice settings, where negative sanctions for noncompliance are the norm. This study engages multiple probation agencies in a Plan-Do-Study-Act process to facilitate local adaptation of the contingency management framework and its deployment in their settings. The research team provides training and structured feedback reports, and cross-site meetings to facilitate sharing of ideas and experiences. Feedback and technical assistance emphasizes adherence to the core principles of contingency management. Encouraging site-specific modifications to the contingency management approach is hypothesized to increase staff buy-in and long-term sustainability of the practice.

### Cross-study measures and outcomes

With the CJ-DATS cooperative providing the common platform for these implementation protocols, NIDA has an opportunity to collect core measures across multiple protocols, and to examine variation across protocols in several key domains. Toward that end, the research teams collect a combination of general and protocol-specific surveys, conduct key informant interviews, and abstract administrative data; when possible, client-level data are collected to inform site decisions about service needs and to provide the criminal justice partner agencies with valuable insights into client outcomes.

Core measures deployed across the medication, HIV, and assessment studies provide the opportunity for data synthesis and harmonization. Baseline core measures provide a snapshot of all of the agencies participating across the cooperative, and yield potential predictor variables for explaining possible site-to-site (or protocol-to-protocol) variations in implementation outcomes. The core measures are collected from line staff, supervisors, and agency leaders (as appropriate) in the participating criminal justice and treatment service provider agencies. These measures include most subscales of the Organizational Readiness to Change survey, including needs/pressures for change (Rowan-Szal et al. 2007; Lehman et al. 2002); resources (staff, training, equipment) (Lehman et al. 2002), staff attributes (Lehman et al. 2002; Broome et al. 2009), and organizational climate. The core measures also include domains from the Survey of Organizational Functioning, including burnout (Garner et al. 2007), perceived organizational support, and leadership (Broome et al. 2009). In addition, the core measures also include domains from the Evidence Based Practice Attitude Scale (Aarons 2004) and baseline measures of interorganizational service coordination (Fletcher et al. 2009).

Centrally, NIDA monitors the progress of each study protocol on a monthly basis, via reports from each of the research centers. These progress reports include the dates marking achievement of key milestones in each protocol. These dates themselves become data for a NIDA internal monitoring process that is based on Chamberlain et al.’s Stages of Implementation Completion (Chamberlain et al. 2011). This provides a structure for measuring site-by-site time-to-completion of each stage of each protocol, the duration of each key stage, and whether each site ultimately progressed through all planned phases of the implementation intervention. This approach is useful for at least three purposes: (a) as a project monitoring tool, to ensure each research grant is achieving adequate and timely progress; (b) to measure the actual duration of each protocol phase as implemented; and (c) as a source of potential predictor measures to include in analyses, for example, to assess whether time-in-phase was related to implementation success or sustainment.

## Discussion

Criminal justice agencies face myriad day-to-day difficulties coordinating communications and service delivery across multiple settings. Tight operating budgets, heavy regulation of operating procedures, and intense public scrutiny of these systems add to the challenges of working in criminal justice environments. Participation in multisite implementation research studies – without an increase in operating budgets – adds additional layers of complexity. The research projects themselves introduce constraints and highlight cross-site variations which require monitoring and documentation, as these may affect study outcomes. For example, there are restrictions on the allowable use of NIH grant funds; NIDA requirements embedded in the conditions of grant award; the need for coordination of multiple study protocols within and across sites; the need for all sites to obtain human subjects approval from local Institutional Review Boards; and a vast and varied array of local mandates that dictate which services or structures are amenable to change and which are fixed by state or Federal statute. A series of comprehensive Federal research protection requirements apply to vulnerable populations, including individuals involved in the criminal justice system. Together, these combine to make NIH-funded research in correctional settings a challenging endeavor.

The US criminal justice system offers myriad opportunities for implementation research on health services issues. Implementation science opens the possibility of reducing drug use, related health problems, and criminal activity by embedding evidence-based addiction treatment and related health services interventions into criminal justice systems. The study protocols described in this collection test four approaches to service and system integration. Findings from these studies are expected to yield scalable implementation strategies that can increase availability, access, delivery, and quality of health care services for offender populations, thereby improving both public safety and public health.

## Authors’ information

The authors are extramural staff at the National Institute on Drug Abuse. There was no external funding for this work.
