# Erratum to: Implementing drug abuse treatment services in criminal justice settings: introduction to the CJ-DATS study protocol series

**DOI:** 10.1186/2194-7899-2-3

**Published:** 2014-03-19

**Authors:** Lori J Ducharme, Redonna K Chandler, Tisha RA Wiley

**Affiliations:** National Institute on Drug Abuse, 6001 Executive Blvd., Rm 5185 MSC 9589, Bethesda, MD 20892-9589 USA

## Correction

This article (Ducharme et al. 2013) was published with the incorrect copyright line; please find the correct copyright line of this article below:

© 2013 The article is a work of the United States Government; Title 17 U.S.C 105 provides that copyright protection is not available for any work of the United States government in the United States; licensee Springer.

This is an open access article distributed under the terms of the Creative Commons Attribution License (http://creativecommons.org/licenses/by/2.0), which permits unrestricted use, distribution, and reproduction in any medium, provided the original work is properly cited.
